# Determination and Pharmacokinetic of Peiminine in Beagle Dogs by UPLC‐MS/MS

**DOI:** 10.1155/ianc/4144078

**Published:** 2026-01-10

**Authors:** Qian Cheng, Xinyu Peng, Xiaotong Li, Xueying Jia

**Affiliations:** ^1^ Innovation Practice Platform for College Student, College of Basic Medicine and Forensic Medicine, Henan University of Science and Technology, Luoyang, Henan, China, haust.edu.cn

**Keywords:** beagle dog, peiminine, pharmacokinetics, UPLC–MS/MS

## Abstract

This study centered on creating and validating a UPLC–MS/MS assay that is both reliable and simple, making it suitable for measuring peiminine levels in the plasma of beagle dogs. The established method was then employed with the ultimate goal of elucidating the pharmacokinetic behavior of the compound. The Acquity UPLC BEH C18 chromatographic column was used for separating peiminine and camptothecin (internal standard, ISTD). A binary mobile phase consisting of acetonitrile and 0.1% formic acid in water was used for gradient elution at 0.4 mL/min. In the multireaction monitoring mode, peiminine and a triple quadrupole mass spectrometer with an electrospray ionization source were utilized to monitor peiminine and the ISTD, and detection was performed by monitoring the following transitions: m/z 430.28 ⟶ 412.25 for peiminine and m/z 349.03 ⟶ 305.09 for the ISTD. Results indicated that the accuracy was around 100%, with both interday precision and intraday (RSD) being less than 10.37%. Additionally, a linear response for peiminine was validated over the range of 1–200 ng/mL, with the LLOQ established at 1 ng/mL. In summary, this study perfectly combined the ultrahigh chromatographic separation ability with the ultrahigh sensitivity, selectivity, and structural analysis ability of mass spectrometry, achieving rapid (2 min), accurate, and ultrasensitive (LLOQ 1 ng/mL) analysis of peiminine in samples. Using the developed method, the pharmacokinetic profile of peiminine was successfully characterized in beagle dogs following oral administration.

## 1. Introduction

The perennial herb *Fritillaria* (Liliaceae) holds a significant place in traditional TCM, with a history spanning over two millennia due to its medicinal benefits [1]. *Fritillaria* is a common TCM which can also be consumed as food in China. Fritillaria alkaloids are the main bioactive components of *Fritillaria* [2]. Peiminine (Figure 1(a)) is the main natural alkaloid compound and the major biologically active component of *Fritillaria thunbergii* Miq, which has good anti‐inflammatory, anticancer, and antiosteoclast effects [3, 4]. The expression levels of particular genes and proteins can be reduced by peiminine in an in vitro setting, thereby inhibiting the differentiation and function of osteoclasts, and the signaling pathways implicated as potential targets of peiminine encompass both NF‐κB and ERK1/2. In ovariectomized mouse models, bone loss can be reduced by peiminine [5]. Peiminine exerts cardioprotective effects on myocardial injury and fibrosis caused by myocardial infarction by inactivating the mitogen‐activated protein kinase pathway [6]. Peiminine can significantly reduce LPS‐induced phosphorylation of AKT and PI3K, thereby alleviating LPS‐induced acute lung injury in mice [7]. Peiminine exerts its effects through distinct molecular mechanisms. The activation of the Nrf2/HO1 signaling pathway underlies its ability to suppress breast cancer progression and mitigate intestinal epithelial apoptosis in murine models of Crohn’s disease. Concurrently, its ability to induce cell cycle arrest at the G0/G1 checkpoint, apoptosis, and autophagy in human osteosarcoma cells is mediated via the ROS/JNK signaling axis [8–10]. Peiminine inhibits the survival rate, colony formation, and metastasis of colorectal cancer cells through the LINC00659/miR‐760 axis, thereby suppressing the development of colorectal cancer [11].

Figure 1The chemical structure of peiminine (a) and camptothecin, ISTD (b).

**Figure:**
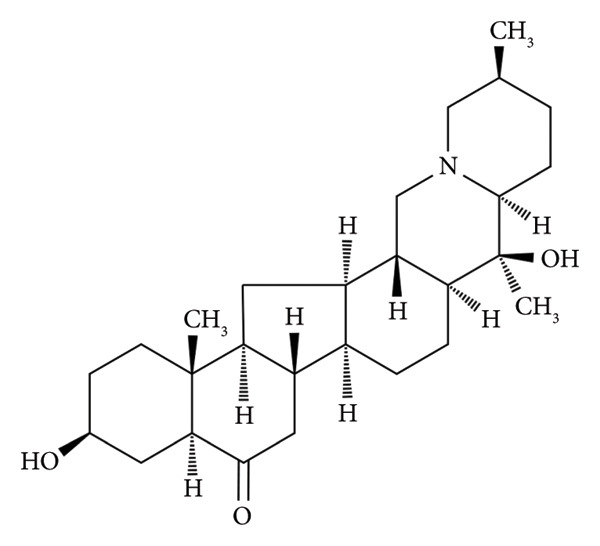
(a)

**Figure:**
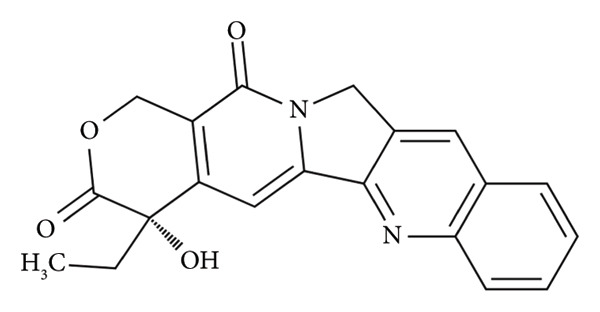
(b)

HPLC–DAD–ELSD methods were used to determine peiminine in total alkaloids of *Fritillaria cirrhosa*, and UPLC–MS methods were used to identify peiminine in the water extract of *Fritillariae thunbergii bulbus* and to investigate their related effects [12, 13]. The study involved the implementation of UPLC–MS/MS as an analytical approach to characterize its pharmacokinetic profile and the determination of peiminine levels in rat plasma [14]. As an analytical technique, UPLC–MS/MS is characterized by its notable speed, sensitivity, and efficient solvent consumption and is a commonly used analytical method for detecting active ingredients and monomers in traditional Chinese herbal medicine [15–17]. The objectives of this work were to develop a sensitive, fast, and practical UPLC–MS/MS method, utilizing camptothecin as the internal standard (ISTD, Figure 1(b)), for quantifying peiminine in plasma, and to subsequently characterize its pharmacokinetics in this animal model.

## 2. Materials and Methods

### 2.1. Chemicals and Drugs

Peiminine and camptothecin (both > 99% pure) were sourced from Chengdu Mansite Biotechnology Co., Ltd. (China). HPLC‐grade methanol and acetonitrile (meeting ACS criteria) were obtained from Kewell Chem LLC (USA), and formic acid was procured from Sigma‐Aldrich (Shanghai) Trading Co., Ltd. (China).

### 2.2. Instruments

The utilized UPLC instrument (Waters Acquity series) featured a sample manager employing a flow‐through needle (SM‐FTN, 1860‐15017), a column manager (CM, 1860‐15043), and a quaternary solvent manager (QSM, 1860‐15018). An electrospray ionization triple quadrupole mass spectrometer (Waters Xevo TQ‐S, Waters Corp., USA) served as the detection system. A vortex mixer (XH‐C, Changzhou Runhua Electric Appliance Co., Ltd., Changzhou, China), ultrapure water equipment (UPR‐II‐10t, Sichuan Youpu Ultrapure Technology Co., Ltd., Chengdu, Sichuan, China), an electronic analytical balance (FA‐2014B, Mettler Toledo Technology (China) Co., Ltd., Shanghai, China), and additional apparatus were also used.

### 2.3. Solution Preparation

A stock solution of peiminine (1 mg/mL) was prepared by first precisely weighing the compound, then transferring it to a 10‐mL volumetric flask, and finally dissolving and diluting to the mark with methanol. Serial tenfold dilutions of this stock with methanol produced standard working solutions at gradient concentrations (100, 10, and 1 μg/mL). The addition of varying concentrations and volumes of standard working solutions to blank beagle dog plasma was used to generate the peiminine calibration curve, at the levels (1, 2, 5, 10, 20, 50, 100, and 200 ng/mL), covering a range from 1 to 200 ng/mL.

The samples spanning three concentration levels (low, medium, and high: 2, 40, and 160 ng/mL, respectively) were generated using the aforementioned method. By diluting the 1‐mg/mL camptothecin stock solution with methanol, a solution of the ISTD at 1000 ng/mL was prepared.

### 2.4. Animal Experiments

Six beagles (8–10 kg) were maintained at the Henan University of Science and Technology animal facility and were supplied under certification SCXK2021 (Hubei)‐0020 by Hubei Yizhicheng Biotechnology Co., Ltd. (Yingcheng, Hubei). Six beagle dogs were housed under a twice‐daily feeding regimen, with water available ad libitum. Before the experiment, animals were subjected to a 12‐h fast (water permitted). All procedures received approval from the Henan University of Science and Technology animal facility (Approval No. 202407004) and were performed in compliance with the NIH Guide for the Care and Use of Laboratory Animals.

Using 0.1% sodium carboxymethylcellulose solution as the vehicle, peiminine was dissolved to achieve 14 mg/mL. Six beagle dogs were orally administered peiminine at 7 mg/kg and then given pure 20 mL of water immediately through oral administration to ensure that the drug entered the body. Plasma was obtained by centrifuging collected blood samples (1.5 mL) at 3000 rpm for 10 min, and the separated plasma was maintained at −20°C, pending analysis. Blood collection occurred at specified intervals: 0.33, 0.67, 1, 1.5, 2, 3, 4, 6, 9, 12, and 24 h after administration.

### 2.5. Pretreatment of Sample

First, the samples were precisely transferred to a tube (1.5‐mL Eppendorf). Next, the addition of 10 μL from the 1000‐ng/mL ISTD working solution was performed. Following brief mixing, 200 μL of acetonitrile was introduced, and the mixture was subsequently vortexed for 2 min. The clear upper layer obtained after centrifugation (10 min at 15, 000*g*) was subsequently pipetted into the autosampler vials for analysis. When UPLC–MS/MS detection was performed, the injection volume was set to 2 µL for analysis.

### 2.6. UPLC–MS/MS Conditions

Employing an Acquity UPLC BEH C18 column (2.1 × 50 mm, 1.7 μm) and an acetonitrile/0.1% aqueous formic acid mobile phase, separation was conducted under the gradient conditions specified in Table 1. Separation was performed at a column temperature of 40°C with a 2‐μL injection volume.

**Table 1:** The UPLC gradient elution program.

Time (min)	Acetonitrile (%)	0.1% formic acid (%)	Flow rate (mL/min)
0–0.5	10	90	0.4
0.5–1.0	90	10	0.4
1.0–1.4	90	10	0.4
1.4–1.5	10	90	0.4
1.5–2.0	10	90	0.4

Mass spectrometry detection adopted the multiple reactions monitoring (MRM) in ESI + mode, and for peiminine, the parent ion transition was at m/z 430.28 and the daughter ion transition at m/z 412.25; for the ISTD, the parent ion transition was at m/z 349.03 and the daughter ion transition at m/z 305.09. The mass spectrometric parameters were as follows: for peiminine, dwell time = 0.146 s, cone voltage = 10 V, and collision energy (CE) = 30 V, and for the ISTD, dwell time = 0.025 s, cone voltage = 10 V, and CE = 20 V.

### 2.7. Method Validation

The contents of methodological validation included a calibration curve, along with assessments of accuracy, specificity, precision, matrix effect (ME), stability and recovery, and the determination of the LLOQ. Method validation for the UPLC–MS/MS assay was conducted in line with the pertinent guidelines of the Chinese Pharmacopoeia (2020 Edition) to ensure its suitability for biosample quantification [18].

#### 2.7.1. Selectivity

Method selectivity was verified to ensure clear distinction of the target analyte and the ISTD from any endogenous or other sample matrix components. To assess method selectivity, six beagle dogs provided the blank plasma samples used in this assessment.

#### 2.7.2. LLOQ and Calibration Curve

The peiminine plasma calibration curve (1, 2, 5, 10, 20, 50, 100, and 200 ng/mL) was treated, and following detection, the respective peak areas for peiminine (As) and the ISTD (Ai) were measured, respectively. A standard curve was generated with the As/Ai ratio plotted against concentration, whereby the lowest concentration point was designated as the LLOQ.

#### 2.7.3. Accuracy and Precision

Three levels of concentrations (2, 40, and 160 ng/mL) QC samples were treated and detected. Precision and accuracy were assessed for both intraday and interday variations. Intraday values were derived from the results obtained within a single day, whereas interday values were obtained from data collected over three consecutive days.

#### 2.7.4. Recovery and ME

Through a comparison of the chromatographic response for the analyte in the processed sample with that in a neat standard at an equivalent concentration, the recovery was determined. ME was assessed by comparing the analyte’s signal in the sample matrix versus its counterpart in a pure standard solution. The recovery and ME were also examined at three concentrations: 2, 40, and 160 ng/mL.

#### 2.7.5. Stability

A stability assessment was conducted on samples across a range from low to high levels. The tested scenarios included short‐term room temperature stability (3 h), refrigerated stability of processed samples (4°C, 12 h), stability after three cycles of freezing and thawing from −20°C to room temperature (25°C), and long‐term frozen stability (−20°C, 4 weeks). The stability results are expressed in terms of RSD (%) and accuracy (%).

### 2.8. Pharmacokinetic Parameter Calculation

To determine the concentration of peiminine, the developed UPLC–MS/MS method was applied to analyze the samples. Data processing was conducted with DAS (Version 2.0) to derive pharmacokinetic parameters. The *T*_max_ and *C*_max_ were recorded as observed values, and the plasma concentration–time curve for peiminine was subsequently generated.

## 3. Results and Discussion

### 3.1. Optimization and Method Development

Because acetonitrile has stronger elution ability and sharper chromatographic peaks, and acetonitrile is a commonly used mobile phase in chromatographic analysis [19, 20], it was chosen to serve as the organic component. The pKa of peiminine is 14.86, which is in an ionic state under acidic mobile phase conditions and is more easily ionized during mass spectrometry detection. Based on the above optimization, acetonitrile and 0.1% formic acid aqueous solution were therefore chosen as the mobile phase for this method. At the same time, peiminine is weakly alkaline, and ESI was applied to MRM in positive ion mode during mass spectrometry detection. The acidic mobile phase is suitable for positive ion detection, and the inclusion of 0.1% formic acid in the mobile phase serves to enhance ionization efficiency. Alkaline compounds are more stable under ESI + conditions.

When selecting the ISTD, comparisons were made between camptothecin, berberine, and tanshinone IIA, and the results showed that camptothecin had a good peak shape and a retention time similar to that of peiminine. Therefore, camptothecin was chosen as the ISTD.

MRM was optimized by prioritizing the selection of parent ions ([M + H]^+^), avoiding adducts, selecting 2–3 characteristic daughter ions, avoiding background interference, and ensuring maximum signal‐to‐noise ratio through stepwise optimization (±5 eV) of CE. After MRM scanning of peiminine and the ISTD, the characteristic figures of parent ions and daughter ions are shown in Figure 2. The daughter ion and parent ion of peiminine were 430.28 and 412.25, respectively; the parent ion and daughter ion of ISTD were 349.03 and 305.09, respectively.

Figure 2The MS2 spectra of peiminine (a) and the ISTD (b).

**Figure:**
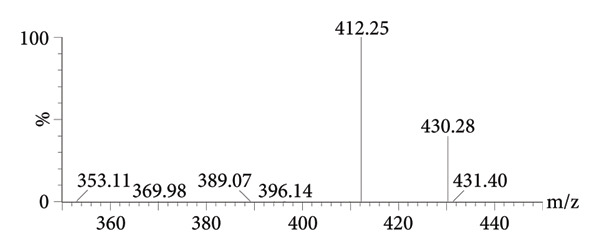
(a)

**Figure:**
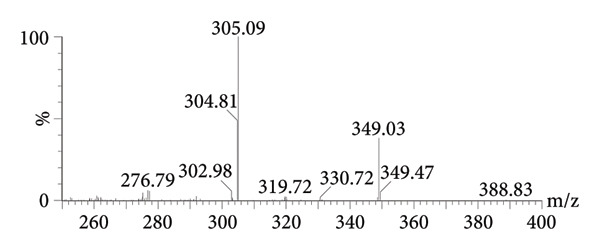
(b)

Protein precipitation was performed on samples employing acetonitrile, which was not only simple and efficient but also satisfied the acceptance criteria for both the recovery and the ME. This one‐step treatment method reduces environmental pollution and meets the requirements of green chemistry [21].

### 3.2. Method Validation

#### 3.2.1. Selectivity

Figure 3 presents the representative chromatograms. It could be seen that the chromatographic peaks of peiminine and the ISTD in beagle dog plasma were well resolved, which indicated that endogenous substances in plasma did not affect the determination. Analysis in positive ion mode showed that peiminine was eluted at 1.17 min, while the ISTD had a retention time of 1.25 min.

Figure 3Representative chromatograms: (a) a beagle blank plasma sample; (b) a blank plasma sample spiked with peiminine (20 ng/mL) and the ISTD; and (c) a beagle dog sample (1.5 h after administration).

**Figure:**
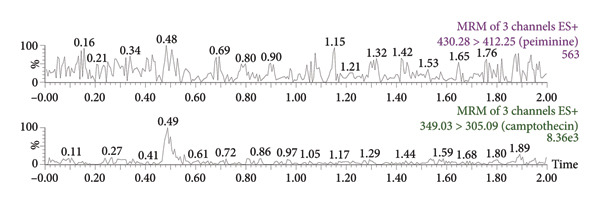
(a)

**Figure:**
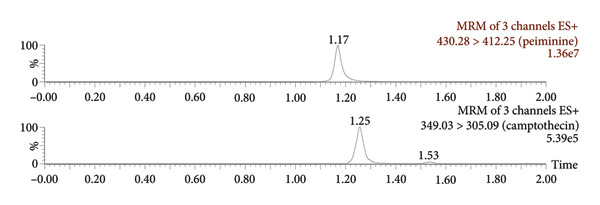
(b)

**Figure:**
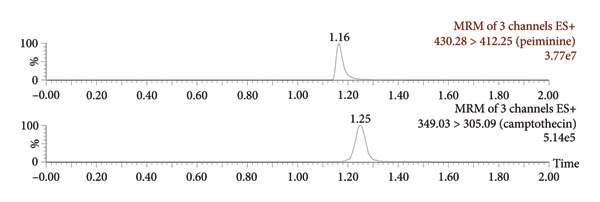
(c)

#### 3.2.2. Calibration Curve and LLOQ

A linear relationship for peiminine was established with the regression equation *y* = 3.58 × 10^−2^ *x* + 5.53 × 10^−2^ (where *x* and *y* represent the plasma concentration and peak area ratio, respectively) and 0.9993 (r). This linearity was demonstrated across the concentration range of 1–200 ng/mL.

#### 3.2.3. Precision and Accuracy

The precision (RSD, %) was below 10.37%, and the accuracy (%) was between 97.17% and 101.50%; all results satisfied the acceptance criteria. According to Table 2, the method demonstrated both accuracy and precision, confirming its reliability for analysis.

**Table 2:** Precision and accuracy of peiminine in beagle dog plasma (*n* = 6, mean ± SD).

Added (ng/mL)	Intraday		Interday	
	RSD (%)	Accuracy (%)	RSD (%)	Accuracy (%)
1	9.21	97.17	8.25	98.39
2	5.70	101.50	5.84	100.44
40	4.31	100.91	4.04	101.31
160	3.48	98.48	3.09	99.38

#### 3.2.4. Recovery and ME

Table 3 shows the results of the recovery and ME. It could be seen that all values of recovery were between 80.96% and 82.48%. Meanwhile, the ME ranged from 98.48%–101.50%. This result indicates that the ME had no appreciable impact on the quantitation of peiminine in the tested plasma matrix.

**Table 3:** Recovery and ME of peiminine in beagle dog plasma (*n* = 6, mean ± SD).

Added (ng/mL)	Recovery (%)	RSD (%)	ME (%)	RSD (%)
2	80.96 ± 1.80	2.22	101.50 ± 5.79	5.70
40	81.63 ± 2.23	2.73	100.91 ± 4.35	4.31
160	82.48 ± 2.44	2.96	98.48 ± 3.43	3.48

#### 3.2.5. Stability

Peiminine demonstrated good stability in plasma samples under the tested experimental conditions, with no significant degradation observed. This conclusion is supported by the accuracy results (94.51%–103.42%) obtained across four different stability conditions, as detailed in Table 4.

**Table 4:** Results of stability of peiminine under four different conditions (*n* = 6).

Added (ng/mL)	Autosampler 4°C, 6 h		Room temperature, 3 h		Three freeze‐thaw		−20°C, 4 weeks	
	RSD (%)	Accuracy (%)	RSD (%)	Accuracy (%)	RSD (%)	Accuracy (%)	RSD (%)	Accuracy (%)
2	4.90	102.75	5.30	100.33	3.21	103.42	5.42	97.58
40	3.01	94.51	3.68	100.21	4.13	98.70	3.24	102.25
160	2.19	101.58	2.88	98.78	2.33	97.66	1.24	98.80

### 3.3. Pharmacokinetic Application

**Figure 4:**
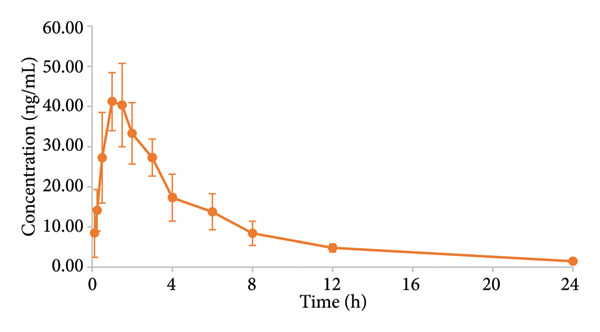
Mean plasma concentration–time curves of peiminine after oral administration to beagle dogs.

UPLC–MS/MS was employed to quantify peiminine in canine plasma following its oral administration. After the beagle dogs were given peiminine 7 mg/kg, the mean plasma concentration–time course of peiminine is illustrated in Figure 4. Table 5 lists the primary pharmacokinetic parameters for peiminine.

**Table 5:** Pharmacokinetic parameters of peiminine after oral administration to beagle dogs (*n* = 6, mean ± SD).

Parameters	Peiminine
*C*_max_ (ng/mL)	43.08 ± 9.45
*T*_max_ (h)	1.17 ± 0.26
*t*_1/2_ (h)	5.04 ± 1.37
MRT_(0 − *t*)_ (h)	5.74 ± 0.27
MRT_(0–*∞*)_ (h)	6.93 ± 0.64
CLz/F (L/h/kg)	29.97 ± 6.06
Vz (L/kg)	221.24 ± 91.81
AUC_(0–*t*)_ (ng·h/mL)	233.23 ± 47.20
AUC_(0–*∞*)_ (ng·h/mL)	241.52 ± 47.49

After oral administration of 7 mg/kg peiminine to six beagle dogs, the *C*_max_ of peiminine reached 43.08 ng/mL at about 1.17 h, and the *t*_1/2_ was about 5.04 h. The absorption of peiminine was relatively rapid in beagle dogs following oral administration, as indicated by the pharmacokinetic results. The apparent distribution volume was relatively large, indicating extensive distribution in tissues. The half‐life of plasma elimination was about 5 h, indicating a faster elimination in beagle dogs.

### 3.4. Study Limitations

The study was performed in healthy beagle dogs under controlled conditions; therefore, the pharmacokinetic profile of peiminine may differ in diseased states or other species, including humans. Additionally, only a single oral dose (7 mg/kg) was tested, so dose‐proportionality and potential nonlinear pharmacokinetics remain uncharacterized, limiting extrapolation to clinical dosing regimens. The use of 0.1% sodium carboxymethylcellulose as a vehicle may also influence drug absorption compared to other formulations.

## 4. Conclusions

In summary, this study perfectly combined the ultrahigh chromatographic separation ability with the ultrahigh sensitivity, selectivity, and structural analysis ability of mass spectrometry, achieving rapid (2 min), accurate, and high‐sensitivity (LLOQ 1 ng/mL) analysis of peiminine in samples. This study successfully employed the validated method to investigate the pharmacokinetic profile of peiminine in beagle dogs after oral dosing.

## Conflicts of Interest

The authors declare no conflicts of interest.

## Author Contributions

Qian Cheng: conceptualization, methodology, inquiry, and drafting the manuscript. Xinyu Peng: investigation, software, and drafting the manuscript. Xiaotong Li and Xueying Jia: formal analysis, methodology, and validation.

## Funding

The authors declare that no specific grant or funding was obtained for this work.

## Data Availability

The corresponding author will provide the underlying research data upon receipt of a justified inquiry.
